# Improving Loanword Identification in Low-Resource Language with Data Augmentation and Multiple Feature Fusion

**DOI:** 10.1155/2021/9975078

**Published:** 2021-04-08

**Authors:** Chenggang Mi, Shaolin Zhu, Rui Nie

**Affiliations:** ^1^School of Computer Science, Northwestern Polytechnical University, Xi'an, China; ^2^College of Software Engineering, Zhengzhou University of Light Industry, Zhengzhou, China; ^3^Chinese Flight Test Establishment, Xi'an, China

## Abstract

Loanword identification is studied in recent years to alleviate data sparseness in several natural language processing (NLP) tasks, such as machine translation, cross-lingual information retrieval, and so on. However, recent studies on this topic usually put efforts on high-resource languages (such as Chinese, English, and Russian); for low-resource languages, such as Uyghur and Mongolian, due to the limitation of resources and lack of annotated data, loanword identification on these languages tends to have lower performance. To overcome this problem, we first propose a lexical constraint-based data augmentation method to generate training data for low-resource language loanword identification; then, a loanword identification model based on a log-linear RNN is introduced to improve the performance of low-resource loanword identification by incorporating features such as word-level embeddings, character-level embeddings, pronunciation similarity, and part-of-speech (POS) into one model. Experimental results on loanword identification in Uyghur (in this study, we mainly focus on Arabic, Chinese, Russian, and Turkish loanwords in Uyghur) showed that our proposed method achieves best performance compared with several strong baseline systems.

## 1. Introduction

**Figure 1:**
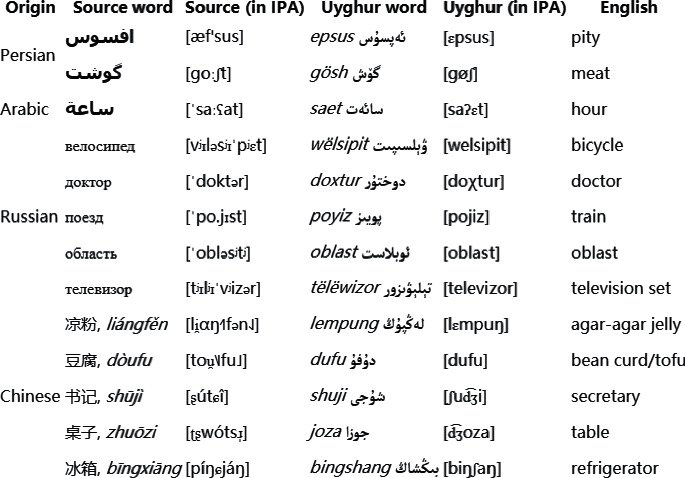
Examples of loanwords in Uyghur2.

Bilingual data play an very important role in cross-lingual natural language processing (NLP) tasks, such as cross-lingual text classification, cross-lingual information retrieval, and neural machine translation. However, bilingual data are often difficult to obtain. Lexical borrowing happens in almost every language; Figure 1 gives several loanwords in Uyghur (the reasons why we choose Uyghur as an example in our study are as follows: (1) there are many loanwords in Uyghur and (2) Uyghur is a low-resource language). If loanwords in low-resource languages can be identified effectively, it will be a novel way to alleviate the data sparseness existing in many cross-lingual NLP tasks.

Loanword identification is a task of finding out loanwords of a specific language (donor language) in texts in another language (receipt language). There are about three kinds of loanword identification methods: (1) rule-based method; (2) statistical-based method; and (3) deep learning-based method. Early studies on loanword identification often based on rules. For example, McCoy and Frank [1] proposed a string similarity-based loanword identification model that relies on the ED algorithm. With the development of machine learning algorithms in NLP area, statistical-based methods are also proposed [2]. In recent years, deep learning algorithm such as bidirectional LSTM and convolutional neural network (BLSTM + CNN) are also used in loanword identification tasks [3]. Due to the lack of generalization ability of rule-based methods and limitation of training data in statistical-based methods, recent studies often combine the rule and statistical features together to improve the model performance effectively [4, 5]. However, almost all of these methods suffer from data sparseness during model training, especially in low-resource settings.

As a common used method to alleviate the data sparseness, data augmentation is one of the most popular methods in this topic. For example, Liu et al. [6] proposed to use a GAN model consisting of two generators and one discriminator to produce meaningful natural language sentences. Motivated by this study, we propose to use a lexical constraint-based data augmentation model to generate more training data for loanword identification. Different from [6], we take the loanwords in training data as a lexical constraint to produce more sentences containing the loanwords.

After investigation, we find that there are two important clues in loanword identification: semantic similarity and pronunciation similarity. To incorporate these two features into one feature, we propose to transfer the semantic similarity as word-level feature and pronunciation similarity as character-level feature. Then, we fuse these two features into one feature. Meanwhile, we incorporate the fusion feature, pronunciation feature, and POS feature into a log-linear RNN to achieve the best performance in loanword identification.

The main contributions of this study are as follows:

- First, a lexical constraint-based data augmentation method is proposed to generate more training data for loanword identification task.
- Second, we incorporate multilevel features, pronunciation similarity feature, and POS feature into a log-linear RNN model to improve the performance of the loanword identification model for low-resource language.
- Third, we conduct an experiment on loanword (Arabic, Chinese, Russian, and Turkish) identification in Uyghur; experimental results show that our proposed model achieves the best performance compared with several strong baseline systems.

The rest of this paper is organized as follows.  Section 2 introduces some recent studies related to our topic. We present details of our proposed method in Section 3. Datasets, settings, and experimental results are described in Section 4. We show the analysis of experimental results in Section 5. In Section 6, we conclude this study and give some possible future directions.

## 2. Related Work

In this section, we present some work related to our study.

### 2.1. Loanword Identification

Lexical borrowing has received relatively little attention in natural language processing area. Tsvetkov and Dyer [7] proposed a morph-phonological transformation model to obtain good performance at predicting donor forms from borrowed forms. Tsvetkov et al. [7] suggested to use the lexical borrowing as a model in an SMT framework to translate OOV words. Gerz et al. [8] analyzed the implication of variation in structural and semantic properties in general language-independent architectures on the language modeling task. Mi et al. [9] used shallow features such as string similarity to detect loanwords in Uyghur. Mi et al. [3] presented a neural network-based loanword identification model that also incorporated several shallow features. However, these methods only trained loanword identification models based on some monolingual corpora. It fails to project donor language and receipt language into one semantic space. The limitation of training data also exists.

### 2.2. Data Augmentation for NLP

The main goal of data augmentation in NLP is to generate additional, synthetic data using the data you have to alleviate the data sparseness during model training [10]. There are several data augmentation methods in NLP area [11]. The first one is lexical substitution which tries to substitute words present in a text without changing the meaning of the sentence [12]. The second one is back translation, which is commonly used in neural machine translation (NMT). Back translation first trains an intermediate system on the parallel data which is used to translate the target monolingual data into the source language. The result is a parallel corpus where the source side is synthetic machine translation output while the target is genuine text written by humans. The synthetic parallel corpus is then simply added to the real bitext in order to train a final system that will translate from the source to the target language [13]. The syntax-tree manipulation has been used in [14]; the idea is to parse and generate the dependency tree of the original sentence, transform it using rules, and generate a paraphrased sentence. Mixup is a simple yet effective image augmentation technique introduced by Zhang et al. [15]. The idea is to combine two random images in a mini-batch in some proportion to generate synthetic examples for training. The most recent data augmentation method is generative model; this kind of method tries to generate additional training data while preserving the class label [16].

### 2.3. Sequence Labeling in NLP

There are two main types of sequence labeling methods in NLP, such as gradient-based methods and search-based methods [17]. As for the probabilistic gradient-based learning methods such as conditional random fields (CRFs) and recurrent neural network (RNN), they have high accuracy because of the exact computation of the gradient and probabilistic information. Nevertheless, those methods have critical drawbacks. First, the probabilistic gradient-based methods typically do not support search-based optimization (search-based learning or decoding-based learning), which is important in sequence labeling problems with emphasis on the learning speed (e.g., for large-scale datasets). In tasks with complex structures, gradient computation is usually quite complicated sometimes and even intractable. This is mainly because dynamic programming for computing gradient is hard to scale for large-scale datasets. On the other hand, the search technique is easier to scale to large-scale datasets. This is because search-based learning is much simpler than gradient-based learning [18–20]—just search the promising output candidates and compare them with the oracle labels and update the weights accordingly. Another category of sequence labeling methods is the search-based learning methods (i.e., decoding-based learning), such as structured perceptron and MIRA. A major advantage of those methods is that they support search-based learning, such that the gradient is not needed and the learning is done by simply searching and comparing the promising output candidates with the oracle labels and updating the model weights accordingly. As a by-product of the avoidance of gradient computation, those methods have faster training speed compared with probabilistic gradient-based learning methods like CRF.

## 3. Method

In previous studies, a large scale of annotated data is used to train a loanword identification model. They treated the loanword detection as a sequence labeling problem. However, the annotated data for loanword identification are very difficult to obtain. So, one of the contributions of this study is the data augmentation for loanword identification. We propose to use a lexical constraint GAN to generate more sentences for loanword identification model training. Another contribution of this paper is the combination of several features for loanword identification model; we introduce three features such as embedding fusion feature (word level and character level), pronunciation similarity feature, and POS feature.

### 3.1. Overall Architecture

Our proposed method includes two parts:

1. Data augmentation for loanword identification.
2. Log-linear RNN-based loanword identification model.

**Figure 2:**
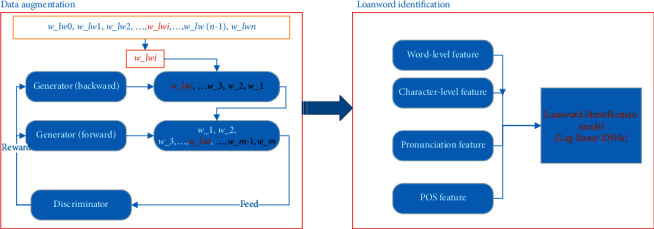
The framework of our proposed model.

To generate more training data for loanword identification, we propose a lexical constraint GAN-based data augmentation model. Recent methods on loanword identification often trained on features such as pronunciation similarity, POS similarity, and so on. However, these kinds of methods usually suffer from data sparseness or lack of semantic knowledge. To overcome this, we introduce a log-linear RNN-based loanword identification model which combines word-level and character-level embedding fusion features, pronunciation similarity, and POS features to predict Arabic, Chinese, Russian, and Turkish loanwords in Uyghur. The main idea of loanword identification in low-resource languages is as follows: we first use the data augmentation model to generate more training data for loanword identification in Uyghur; then, several features such as word- and character-level embedding features, pronunciation similarity, and POS features are proposed to build a multiple feature fusion-based loanword identification model (Figure 2).

### 3.2. Data Augmentation for Loanword Identification

Recent studies on loanword identification task often suffer from limitation of training data. In this study, we propose to use a lexical constraint GAN to generate more annotated data for the loanword identification task. As an extension of traditional GAN, our data augmentation model also includes two main parts: a generator and a discriminator. The difference is that we use two generators and a discriminator to build the data augmentation model for low-resource loanword identification. We introduce the details of our proposed model in this section.

#### 3.2.1. Generators

We follow the work of [6] and extend the backward and forward generators to adapt to the loanword identification task. In our study, we use the loanwords of a specific language as the lexical constraint to generate more training data. Similar to [6], given a loanword, the backward generator takes it as the sentence's starting point and generates the first half sentence backwards. Then, the sequence produced by the backward generator is reversed and fed into the forward generator. It then learns to generate the whole sentence with the aim of fooling the discriminator.

We can define the backward generator *G*_*θ*_^(*bw*)^ as

(1) $$\begin{array}{c} P_{\theta }^{\left(bw\right)}\left(s_{<c}|w_{lw}\right)=\prod_{i=1}^{lw-1}P_{\theta }^{\left(bw\right)}\left(w_{lw-i}|w_{lw},\ldots ,w_{lw-i+1}\right), \end{array}$$

where *w*_*lw*_ denotes a given loanword and *l* indicates the length of generated training sentence. The generated sentence is *s*=*w*_1_*w*_2_,…, *w*_*lw*_,…, *w*_*l*_. The backward generator generates the first half of the sentence, while another half of sentence is generated by the forward generator. *θ* and *θ*′ are parameters of the backward and forward generators.

The generator of the entire sentence can be defined as

(2) $$\begin{array}{c} G\left(s||w_{c}|;|\theta |,|\theta^{\prime }\right)=P_{\theta }^{\left(bw\right)}\left(s_{<c}|w_{lw}\right)P_{\theta^{\prime }}^{fw}\left(s_{<c}|s_{1:lw}\right), \end{array}$$

where *P*_*θ*_^(*bw*)^(*s*_<*c*_*|w*_*lw*_) and *P*_*θ*′_^*fw*^(*s*_<*c*_*|s*_1:*lw*_) are descripted as above.

The two generators have the same structure but have distinct parameters. To improve the coherence of the constrained sentence, we employ an LSTM-based language model with dynamic attention mechanism (called attRNN-LM) as generator.

#### 3.2.2. Discriminator

Another important component in our proposed method is the discriminator, which takes sentence pairs as input and distinguishes whether a given sentence pair is real or generated. It guides the joint training of two generators by assigning proper reward signals. This module can be a binary classifier or a ranker. Following previous methods [21], we use Text-CNN as the discriminator which outputs a probability indicating whether the input is generated by humans or machines in the experiment.

#### 3.2.3. Data Augmentation Model

To train the data augmentation model effectively, we first pretrain the backward and forward generators by standard MLE loss. Different from [6], we sample a loanword in our loanword list as the lexical constraint rather than select it randomly. Then, we use two generators and the lexical constraint to generate the training sentence. The discriminator is trained based on real sentence as positive sample and sentences generated by generators as negative samples. The discriminator's output is the probability that the generated sentence is written by humans. We use the discriminator's output as the reward to encourage the two generators to work together to generate sentences which are indistinguishable from human-written sentence. To make the training stable and prevent the perplexity value skyrocketing, we apply teacher forcing to give the generators access to the gold-standard targets after each policy training step.

### 3.3. Multiple Feature Fusion-Based Loanword Identification

Loanword identification can be defined as a sequence labeling problem. However, different from a traditional sequence labeling task, loanword identification task can apply some additional knowledge such as semantic similarity, pronunciation similarity, and POS tagging. As the data augmentation can provide us more annotated data for model training, we propose to use a deep neural network model to identify loanword in low-resource settings. The principle feature we used is the fusion of word- and character-level features, which combines the word relation and pronunciation similarity in loanword identification. We also incorporate external features such as pronunciation similarity and POS information into our method. In this section, we first describe features used in our proposed method and then define the details of the loanword identification method.

#### 3.3.1. Features

We use three kinds of features in our proposed method: the fusion feature, pronunciation similarity, and POS feature.

*Fusion Feature*. In loanword identification task, word co-occurrence often plays a very important role. For example, in the English sentence “Tiananmen square is the most famous tourist destination in Beijing,” the Chinese loanword “Tiananmen” is most related to the Chinese loanword “Beijing.” In previous work, word embedding can capture word similarity and word relations with other words in a sentence. Therefore, we apply self-attention to obtain word embedding in our study. The most important advantage of the self-attention is that it can model dependencies between words.

We use the dot-product attention in this study:

(3) $$\begin{array}{c} \text{DotAtt}\left(Q,K,V\right)=\text{softmax }\left(QK^{T}\right)V, \end{array}$$

where **Q**, **K**, **V** are query, key, and value vectors, respectively. It should be noted that the self-attention was obtained without scaling. We set

(4) $$\begin{array}{c} Q=K=V=x_{t}^{w}, \end{array}$$

and at time step *t*, the word embedding at time *t* based on self-attention can be defined as

(5) $$\begin{array}{c} h_{t}^{wl}=\text{DotAtt}\left(x_{t}^{w},x_{t}^{w},x_{t}^{w}\right). \end{array}$$

The most important feature in loanword identification task is the pronunciation similarity between the word in receipt language and its corresponding word in donor language. As convolutional neural networks (CNNs) have been proven to capture the character-level information in NLP tasks, CNNs can process the sequences in the current receptive filed akin to the attention mechanism [22]. Meanwhile, we also use max pooling to capture character-level features. The way we use CNN in our proposed method can be defined as

(6) $$\begin{array}{c} \text{Conv}\left(x_{t}^{c}\right)=\text{Mask}\left(x_{t}^{c}\right)*U. \end{array}$$

We follow the study of [23] and use a CNN with a redundant position of input sequences masked to extract the character-level features. *U* is the filter width *k* set as 3. The convolution operation is denoted with *∗*, and the padded position of input sequences is set as 0.

Max means a max pooling operation. We use it to capture the significant features assigned with the highest value for a given filter. Therefore, in the time step *t*, the character-level representation from local view is obtained as

(7) $$\begin{array}{c} h_{t}^{cl}=\text{Max}\left(\text{Conv}\left(x_{t}^{c}\right)\right). \end{array}$$

**Figure 3:**
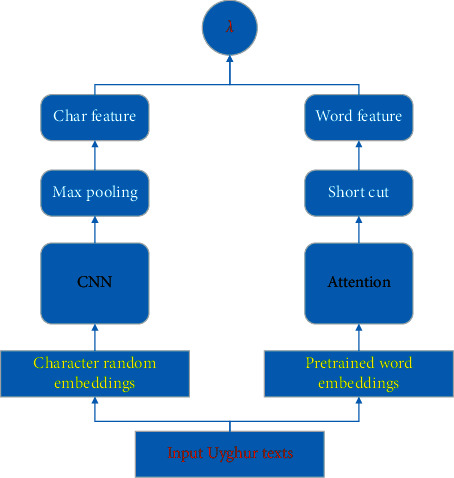
The multilevel feature fusion method used in our proposed loanword identification model. Character embeddings and word embeddings are taken as input for the feature selection layer.

To fuse the word-level and character-level features together, we propose to concatenate two features with automatic adjustment (Figure 3). The final fusion representation can be defined as

(8) $$\begin{array}{c} Z=\lambda_{1}h_{t}^{wl}+\lambda_{2}h_{t}^{cl}, \end{array}$$

where *h*_*t*_^*wl*^ and *h*_*t*_^*cl*^ are word-level and character-level features, respectively, and *λ*_1_ and *λ*_2_ are corresponding parameters.

*Pronunciation Similarity Feature*. Intuitively, we find that a loanword often has a similar pronunciation with its corresponding donor word. A sample method to detect loanwords is to use a string similarity algorithm to compute the string similarity scores between the candidate loanword and a list of words in donor language. Then, we rank the scores and take the word with the best score as the donor word. In loanword identification task, we first transform donor and receipt language texts into a same writing system. For example, in Chinese loanword identification in Uyghur, we first convert these two language texts into Latin. Then, we apply the most commonly used string similarity algorithm—minimum edit distance (MinED)—in our loanword identification task.

(9) $$\begin{array}{c} h_{\text{med}}\left(lw,acrt,u\right)\\ =\overset{l_{acrt}}{\underset{j=0}{\sum }}\overset{l_{u}}{\underset{i=0}{\sum }}Pr\;\left(lw_{i}|\text{med }\left(u_{i},{\text{acrt}}_{j}\right)\right), \end{array}$$

where *l*_**acrt**_ and *l*_**u**_ are lengths of donor word list and receipt word list, respectively, acrt and *u* represent donor languages (Arabic, Chinese, Russian, and Turkish) and receipt language (in this study indicates Uyghur), *lw*_*i*_ is the loanword label of the *i*th receipt word, and med(*u*_*i*_, acrt_*j*_) is the minimum edit distance of two words. To adapt the loanword identification task, we first conduct text normalization on all datasets, which transform a text into a canonical (standard) form. Then, we carry on morphological segmentation on morphologically rich languages, such as Uyghur, Russian, and Turkish.

*POS Feature*. As loanwords are often nouns, we propose a part-of-speech (POS) feature to further constrain the loanword identification model. We first pretrain POS tagging models for donor languages and receipt language. Considering both the language resource and performance, we select CRF as the framework of POS tagging model. As POS models are ready, if a word in receipt and its corresponding candidate donor word are all nouns, we set the POS features as 1.

#### 3.3.2. Loanword Prediction Model

Log-linear models play a considerable role in statistics and machine learning. The most important reason we chose the log-linear model as the basic framework of our proposed loanword prediction model was because features can be easily added into it. Additionally, the log-linear model has been widely used in NLP tasks such as SMT and NMT.

To adapt the loanword prediction task and include rich features such as BiLSTM, POS, and semantic feature into the model, we use log-linear RNNs [24] as the basic framework in our task. Log-linear RNN is similar to a RNN model. It allows a more general form of input to the network at each time step; that is , instead of allowing only the latest symbol *x*_*t*_ to be used as input, along with the condition *C*, it now allows an arbitrary feature vector *ψ*(*C*, *x*_1_, *x*_2_, .., *x*_*t*−1_, *x*_*t*_) to be used as input; this feature vector is of fixed dimensionality |*ψ*| and allows it to be computed in an arbitrary (but deterministic) way from the combination of the currently known prefix *x*_1_, *x*_2_, .., *x*_*t*−1_, *x*_*t*_ and the context *C*. This is a relatively minor change, but one that usefully expands the expressive power of the network.

The hidden state at time *t* in our loanword identification task can be defined as

(10) $$\begin{array}{c} p_{\theta ,t}\left(x\right)\\ \propto b\left(C,x_{1},x_{2},\ldots ,x_{t-1},x_{t}\right)\\ \cdot \;\exp\left(a_{\theta ,t}^{T}\phi \left(C,x_{1},x_{2},\ldots ,x_{t-1},x_{t}\right)\right). \end{array}$$

We assume that we have a priori fixed a certain background function *b*(*C*, *x*_1_, *x*_2_,…, *x*_*t*−1_, *x*_*t*_) and also defined *M* features defining a feature vector *ϕ*(*C*, *x*_1_, *x*_2_,…, *x*_*t*−1_, *x*_*t*_) of fixed dimensionality *ϕ*(*C*, *x*_1_, *x*_2_,…, *x*_*t*−1_, *x*_*t*_).

Therefore, the loanword label of *t*+1 word *x*_*t*+1_ can be defined as

(11) $$\begin{array}{c} x_{t+1}∼p_{\theta ,t}\left(\cdot \right). \end{array}$$

During training of our proposed loanword identification model, we use the cross-entropy loss to optimize the performance of our model [25].

## 4. Experiments

In this section, we evaluate the effectiveness of our proposed method.

### 4.1. Data

To fully evaluate the effectiveness of our proposed model, we conduct Arabic, Chinese, Russian, and Turkish loanword identification in Uyghur. The datasets used in our experiments are listed in Table 1. We crawl these corpora from the Internet. Then, we annotate a small part with loanword label by hands. In all texts, we assure that each sentence includes at least one loanword.

To train the data augmentation model, we also collect some monolingual data from Internet for each language (Table 2).

### 4.2. Settings

#### 4.2.1. Data Augmentation

We train the data augmentation model on datasets described in Table 2. We set the same hyperparameters for forward and backward generators. All generators include 2-layer char-level LSTMs with 1024 hidden units. The dimension of word embeddings is set to 1024; the batch size, dropout rate, threshold of element-wise gradient clipping, and initial learning rate of Adam optimizer are set to 128, 0.5, 5.0, and 0.001; layer normalization is also applied. We set both backward and forward generators to one layered word-level LSTM with 1024 hidden units when training on datasets described in Table 2. For the hyperparameters of the discriminator, the filter window size is set to be 3, 4, 5, 6, and 7, and the kernel number of each filter is 512. We set the batch size as 64 and the number of iterations as 5000.

#### 4.2.2. Loanword Identification

We implemented the log-linear RNNs by ourselves. We also developed the extended version of edit distance algorithm to adapt the loanword identification task. For the POS feature, we first pretrained a Uyghur POS tagging model; then, we tagged all Uyghur sentences based on this model.

We compared our method with several strong baseline systems: Rule [1], CRF [2], BLSTM-CNN [3], and ClEmbedding [4].

### 4.3. Results on Data Augmentation

Results on data augmentation and size of training data can be found in Tables 3 and 4, respectively.

### 4.4. Results on Loanword Identification

The results on loanword identification on different methods can be found in Table 5.

## 5. Analysis

Table 3 presents experimental results on data augmentation for loanword identification. We can find that our proposed lexical constraint method achieves the best performance compared with other strong baseline systems in all evaluation metrics. The most important reason is that our method guarantees the fluency and semantic consistency of generated sentence at the same time.  Table 4 shows the size of Uyghur sentence (with loanword in different donor languages) generated by our proposed data augmentation model. For loanwords in different donor languages, we obtain the largest Uyghur datasets with Turkish loanwords; one possible reason is that Uyghur and Turkish are closely related. We obtain the fewest sentences with Arabic; it is because Uyghur and Turkish have very different grammar and syntax.

The first part in Table 5 describes experimental results on different methods with the original training data. We found that the CRF and rule-based model outperform BLSTM-CNN method; one possible reason is the limitation of annotated data. Because the ClEmbedding model can exploit semantic information obtained from monolingual data, the ClEmbedding model achieves slightly better results compared with the CRF and rule-based model. Compared with other baseline models, our method incorporates word-level and character-level features pretrained from monolingual corpora into one model; therefore, our method achieves best results, but the improvement is not significant. This is because our method also suffers from data sparseness during model training.

The second part of Table 5(with (+)) presents loanword identification results on different methods with our generated training data (data augmentation). We can find that the generated training data improve all baseline models significantly. The CRF-based model has the ability of generalization, but the data sparseness still weakens the loanword identification performance significantly. The BLSTM-CNN + method also achieves better performance compared with the BLSTM-CNN. Both CRF+ and BLSTM-CNN + benefit from data augmentation. Although ClEmbedding + relies on monolingual data, it also obtains performance improvements due to loanword identification results are added. Our proposed method incorporates RNN features and external features into one model, so it achieves the best performance among all baseline systems.

Table 6 presents results on different features in our proposed method (we take Turkish and Chinese loanword identification as examples). We find that models with all features achieve best performance in both Turkish and Chinese loanword identification tasks. As for single feature, the fusion feature is more important than others; one possible reason is that the fusion feature combines word-level and character-level features at the same time. Except the fusion feature, pronunciation similarity feature outperforms other features because the pronunciation similarity is the most intuitive feature in loanword identification task. Although the POS cannot achieve comparative performance with others, we find that the combination features with POS always outperform others.

In Table 5, we describe results on different donor languages. We can easily find that our method achieves best performance on Turkish loanword identification task. One important reason is that Turkish and Uyghur belong to the same language family, and they share much vocabulary and grammar compared with other donor languages. Our model also achieves better results on Russian loanword identification than Chinese and Arabic; one possible reason is that Russian has a deep influence on Uyghur, and Uyghur is sometimes written in a Cyrillic alphabet, which is the basic writing system in Russian. Because people who can speak Uyghur can often speak Chinese fluently, Chinese has a significant impact on Uyghur. Although Uyghur and Arabic share the same writing system, two languages belong to different language families. So, Arabic loanword identification achieves the worst performance.

## 6. Conclusion

The main goal of this study is to improve the performance of loanword identification for low-resource language. Our contribution includes two parts: (1) data augmentation for loanword identification and (2) loanword identification based on multiple feature fusion. In particular, data augmentation alleviates the data sparseness occurring in the loanword identification model training; we optimize the loanword identification model by introducing several features such as fusion feature of word- and character-level embeddings, pronunciation similarity, and POS feature into one model based on a log-linear RNN. To evaluate the effectiveness of our proposed method, we conduct experiments on several baseline models. Experiments show that our proposed loanword identification method achieves the best performance.

In our future work, we plan to improve the robustness of the loanword identification model by generating more diverse training data and incorporating richer contextual information into it.

## Figures and Tables

**Table 1:** Size of datasets.

Data type	Size			
	Arabic	Chinese	Russian	Turkish
Sentences	100, 780	125, 085	143, 290	132, 500
Loanwords	690	2,450	1,274	2,009

**Table 2:** Size of monolingual data.

Languages	Uyghur	Arabic	Chinese	Russian	Turkish
Size (words)	0.32	1.05 B	1.70 B	1.14 B	1.49 B

**Table 3:** Evaluation of data augmentation methods.

Donor	Metrics	B/F-LM	BF-MLE	Ours
Arabic	BLEU-4	0.15	0.15	0.21
	Self-BLEU	64.32	64.58	63.46
	TER	66.19	66.44	65.82

Chinese	BLEU-4	0.16	0.17	0.23
	Self-BLEU	64.05	64.30	63.78
	TER	64.23	65.02	63.98

Russian	BLEU-4	0.18	0.18	0.23
	Self-BLEU	62.76	63.05	62.64
	TER	63.69	63.92	63.45

Turkish	BLEU-4	0.19	0.20	0.25
	Self-BLEU	62.51	62.86	62.18
	TER	62.46	63.14	62.04

**Table 4:** Size of training data generated in data augmentation (Uyghur sentences).

Lang	Arabic	Chinese	Russian	Turkish
Size	302, 480	325, 790	314, 208	336, 852

**Table 5:** Loanword identification experimental results on different methods.

Donor	Model	Loanword identification results (%)					
		*P*	*P*(+)	*R*	*R*(+)	*F*1	*F*1 (+)
Russian	Rule (+)	72.04	72.89	69.31	70.18	70.65	71.28
	CRF (+)	71.63	72.45	67.28	68.15	69.39	70.23
	BLSTM-CNN (+)	71.45	72.26	70.50	71.31	70.97	71.78
	ClEmbedding (+)	73.12	73.94	71.84	72.62	72.47	73.27
	Ours (+)	74.80	75.62	73.64	74.20	74.22	74.90

Arabic	Rule (+)	69.05	69.84	68.17	69.02	68.61	69.43
	CRF (+)	69.83	70.65	67.42	68.29	68.60	69.45
	BLSTM-CNN (+)	68.70	69.52	69.85	70.67	69.27	70.09
	ClEmbedding (+)	72.95	73.76	72.03	72.85	72.49	73.30
	Ours (+)	73.91	74.62	72.35	73.06	73.12	73.83

Turkish	Rule (+)	72.02	72.86	69.87	70.50	70.93	71.66
	CRF (+)	71.46	72.29	69.02	69.95	70.22	71.10
	BLSTM-CNN (+)	71.25	72.04	70.43	71.18	70.84	71.61
	ClEmbedding (+)	72.96	73.64	73.08	73.85	73.02	73.74
	Ours (+)	75.24	76.09	74.36	75.14	74.80	75.61

Chinese	Rule (+)	70.32	71.13	69.77	70.58	70.04	70.85
	CRF (+)	70.85	71.64	69.24	70.05	70.04	70.84
	BLSTM-CNN (+)	70.58	71.34	69.98	70.79	70.28	71.06
	ClEmbedding (+)	71.67	72.48	71.35	72.14	71.51	72.31
	Ours (+)	74.30	75.07	72.88	73.95	73.58	74.51

**Table 6:** Loanword identification results on different features (Turkish and Chinese loanword identification as examples).

Donor	Feature(s)	Loanword identification results (%)					
		*P*	*P*(+)	*R*	*R* (+)	*F*1	*F*1 (+)
Turkish	+fusion	74.14	74.95	73.28	74.16	73.71	74.55
	+pronun	73.96	74.68	73.02	73.94	73.49	74.31
	+pos	72.54	73.36	72.25	73.07	72.39	73.21
	+fusion, pronun	73.40	74.20	72.64	73.40	73.02	73.80
	+fusion, pos	74.63	75.42	73.70	74.52	74.16	74.97
	+pronun, pos	74.25	75.06	73.45	74.24	73.85	74.65
	+all	75.24	76.09	74.36	75.14	74.80	75.61

Chinese	+fusion	73.15	73.94	71.74	72.56	72.44	73.24
	+pronun	72.76	73.52	71.32	72.16	72.03	72.83
	+pos	71.30	72.09	70.58	71.25	70.94	71.67
	+fusion, pronun	72.43	73.25	71.02	71.84	71.72	72.54
	+fusion, pos	73.61	74.40	72.26	73.02	72.93	73.70
	+pronun, pos	73.25	74.03	71.97	72.89	72.60	73.46
	+all	74.30	75.07	72.88	73.95	73.58	74.51

## Data Availability

The data used to support the findings of this study are available from the corresponding author upon request.
